# Supplementary material for: Proteomic Analysis of Zeb1 Interactome in Breast Carcinoma Cells
Source: Molecules. 2021 May 24;26(11):3143. doi: 10.3390/molecules26113143 (PMC8197395; doi:10.3390/molecules26113143)
Supplement: Supplementary file 1 [file molecules-26-03143-s001.zip › molecules-1049562-supplementary.pdf]

Accession	Description	Sequence length	Unused score C1	C2	C3	24,1	24,2	24,3	72,1	72,2	72,3	Sc summ C1	C2	C3	24,1	24,2	24,3	72,1	72,2	72,3
sp|Q14980|NUMA1_HUMAN	Nuclear mitotic apparatus protein 1	2115	87,79	21,34	12,27	12,82	91,94	41,87	11,2	4,3	31,99	565	105	54	57	640	234	52	26	155
sp|P06748|NPM_HUMAN	Nucleophosmin	294	14,11	2,01	2	2,47	18	2			2	278	8	10	9	172	8			8
sp|P17844|DDX5_HUMAN	Probable ATP-dependent RNA helicase DDX5	614	37,85	17,95	12,29	18,95	45,74	15,09	32,63	20,44	12,29	254	86	125	104	236	160	184	134	101
sp|P43243|MATR3_HUMAN	Matrin-3	847	26,09	8,39			29,74	2,95	4,36	6,37	14,48	221	39			175	14	18	40	75
sp|Q08211|DHX9_HUMAN	ATP-dependent RNA helicase A	1270	31,36		2,03		42,29				3,06	213		9		282				12
sp|Q9P258|RCC2_HUMAN	Protein RCC2	522	21,14	19,32	26,28	15,24	23,39	20,83	30,3	15,52	12,16	145	97	171	87	220	139	235	109	70
sp|Q92841|DDX17_HUMAN	Probable ATP-dependent RNA helicase DDX17	729	8,37	7,4	24,21	9,42	9,26	27,73	16	9,91	20,71	138	62	129	84	109	147	147	106	111
sp|P52272|HNRPM_HUMAN	Heterogeneous nuclear ribonucleoprotein M	730	21,25	2,09			31,14	2			10,11	134	9			173	9			47
sp|P26599|PTBP1_HUMAN	Polypyrimidine tract-binding protein 1	531	11,22	14,41	12,13	10,32	11,24	15,52	15,31	4,27	13,01	133	75	76	63	105	108	108	32	85
sp|P55265|DSRAD_HUMAN	Double-stranded RNA-specific adenosine deaminase	1226	14,45	2,78	2	3,56	19,33	4			6,11	123	7	8	14	110	18			31
sp|P61978|HNRPK_HUMAN	Heterogeneous nuclear ribonucleoprotein K	463	16,81	13,1	12,07	12,22	18,99	11,13	17,53	9,82	8,03	117	79	64	77	146	74	102	56	59
sp|P10809|CH60_HUMAN	60 kDa heat shock protein, mitochondrial	573	10,05		4		16					116		13		134				
sp|P11387|TOP1_HUMAN	DNA topoisomerase 1	765	15,3				15,7					110				113				
sp|Q06830|PRDX1_HUMAN	Peroxiredoxin-1	199	13,54	18,7	22,29	25,15	30,25	17,66	22,24	26,39	18,16	107	136	153	183	213	141	149	245	134
sp|Q9NR30|DDX21_HUMAN	Nucleolar RNA helicase 2	783	19,99				25,95					107				126				
sp|P31943|HNRH1_HUMAN	Heterogeneous nuclear ribonucleoprotein H	449	13,08	10,95	8,07	8,34	12,17	10	14,88	10	13,5	102	63	46	47	83	83	91	87	113
sp|P68104|EF1A1_HUMAN	Elongation factor 1-alpha 1	462	11,31	14,17	14	15,18	11,7	14,81	12,54	4,63	12,68	95	100	85	107	97	142	91	58	100
sp|P05388|RLA0_HUMAN	60S acidic ribosomal protein P0	317	11,34				10					79				80				
sp|P68363|TBA1B_HUMAN	Tubulin alpha-1B chain	451	10,89	3,55	2,06	8,26	14,42	5,37		9,55	2	78	16	10	34	121	29		53	7
sp|Q00839|HNRPU_HUMAN	Heterogeneous nuclear ribonucleoprotein U	825	11,4				7,11					75				36				
sp|Q02878|RL6_HUMAN	60S ribosomal protein L6	288	10,86				12					74				88				
sp|P19338|NUCL_HUMAN	Nucleolin	710	10,28				8					68				60				
sp|P49411|EFTU_HUMAN	Elongation factor Tu, mitochondrial	452	10,16	5,32	8,07	7,51	14,05	3,89	7,1		4,07	66	28	48	35	92	16	30		17
sp|P60709|ACTB_HUMAN	Actin, cytoplasmic 1	375	5,31	8,51	18,11	13,59	12,34	8,98	13,45	7,59	11,36	58	67	140	90	90	69	94	64	73
sp|O60506|HNRPQ_HUMAN	Heterogeneous nuclear ribonucleoprotein Q	623	8,01									51								
sp|P36873|PP1G_HUMAN	Serine/threonine-protein phosphatase PP1-gamma cataly	323	1,17				4,14					51				19				
sp|P25705|ATPA_HUMAN	ATP synthase subunit alpha, mitochondrial	553	7,19				8,05					50				34				
sp|P62888|RL30_HUMAN	60S ribosomal protein L30	115	6				6					50				44				
sp|P62424|RL7A_HUMAN	60S ribosomal protein L7a	266	9,52				8,76					49				54				
sp|P16403|H12_HUMAN	Histone H1.2	213	7,57				6,82					48				51				
cont|0050|R9VYW2_LAMGL	VHH variable region [Lama glama (contaminant)]	121	2	2	2	2		2			2	47	30	35	33		71			68
sp|Q12905|ILF2_HUMAN	Interleukin enhancer-binding factor 2	390	8,99				10,05					46				55				
sp|P14866|HNRPL_HUMAN	Heterogeneous nuclear ribonucleoprotein L	589	5,45	8	6	4,03	8,05	8,02	18,36		6,96	45	61	35	16	48	50	110		55
sp|P62906|RL10A_HUMAN	60S ribosomal protein L10a	217	4,18				8,13					45				58				
sp|P49756|RBM25_HUMAN	RNA-binding protein 25	843	6,37				6	1,26			1,49	44				33	5			5
sp|P11142|HSP7C_HUMAN	Heat shock cognate 71 kDa protein	646	5,03		8		5,27	6,17	4,52		8,57	42		35		26	24	19		33
sp|P18124|RL7_HUMAN	60S ribosomal protein L7	248	5,05				8,1					41				51				
sp|P36578|RL4_HUMAN	60S ribosomal protein L4	427	7,04				11,47					41				75				
sp|P78527|PRKDC_HUMAN	DNA-dependent protein kinase catalytic subunit	4128	9,09	2,47	4,28		13,84	12,48	9,53		5,06	38	14	18		55	50	34		15
sp|Q13148|TADBP_HUMAN	TAR DNA-binding protein 43	414	6	2	2	4	6,06	2			4	38	9	9	19	65	12			29
sp|P22087|FBRL_HUMAN	rRNA 2'-O-methyltransferase fibrillarin	321	7,98				20					37				104				
sp|Q14498|RBM39_HUMAN	RNA-binding protein 39	530	6,12				4,06					37				19				
sp|P62805|H4_HUMAN	Histone H4	103	5,65	6	6	8	8,39	5,7		9,85	6	35	21	23	42	68	33		51	25
sp|P51991|ROA3_HUMAN	Heterogeneous nuclear ribonucleoprotein A3	378	4,04	2	4,07	2,81	6		6,24	4		34	9	20	24	30		49	25	
sp|P16402|H13_HUMAN	Histone H1.3	221	0,7									32								
sp|P57721|PCBP3_HUMAN	Poly(rC)-binding protein 3	371	4		2,35	3,71						30		10	16					

sp|Q07020|RL18_HUMAN	60S ribosomal protein L18	188	4,58				4,17					29				29				
sp|P04406|G3P_HUMAN	Glyceraldehyde-3-phosphate dehydrogenase	335	5,89				12,02					27				96				
sp|Q9UMS4|PRP19_HUMAN	Pre-mRNA-processing factor 19	504	5,91				7,16					27				37				
sp|P62241|RS8_HUMAN	40S ribosomal protein S8	208	4,9									26								
sp|P30050|RL12_HUMAN	60S ribosomal protein L12	165	4									24								
sp|O75400|PR40A_HUMAN	Pre-mRNA-processing factor 40 homolog A	957	4				4,11					23				22				
sp|O00571|DDX3X_HUMAN	ATP-dependent RNA helicase DDX3X	662	3,65	2,55		2,7		7,77			6,06	21	14		13		40			27
sp|P50914|RL14_HUMAN	60S ribosomal protein L14	215	4				4					21				34				
sp|P62917|RL8_HUMAN	60S ribosomal protein L8	257	4									21								
sp|Q9BWF3|RBM4_HUMAN	RNA-binding protein 4	364	4				4				2	21				18				12
sp|Q92945|FUBP2_HUMAN	Far upstream element-binding protein 2	711	3,37	8,08	4	1,52	5,31	17,19	9,09	9,03	16,11	19	34	18	6	28	109	45	37	92
sp|P0DN76|U2AF5_HUMAN	Splicing factor U2AF 35 kDa subunit-like protein	240	2									19								
sp|P39748|FEN1_HUMAN	Flap endonuclease 1	380	3,15									19								
sp|P42766|RL35_HUMAN	60S ribosomal protein L35	123	2									19								
sp|P61313|RL15_HUMAN	60S ribosomal protein L15	204	2,24				4,97					19				28				
sp|O60814|H2B1K_HUMAN	Histone H2B type 1-K	126	3,66			2,04	5,95					18			10	37				
sp|P16104|H2AX_HUMAN	Histone H2AX	143	2,28				8					18				68				
sp|P61247|RS3A_HUMAN	40S ribosomal protein S3a	264	3,51									18								
sp|Q96I24|FUBP3_HUMAN	Far upstream element-binding protein 3	572	4,4	5,15	6	3,06		6,06	5,84	4	4,24	17	24	25	21		27	31	19	29
sp|P12956|XRCC6_HUMAN	X-ray repair cross-complementing protein 6	609	3,03							3,62		17							21	
sp|Q12906|ILF3_HUMAN	Interleukin enhancer-binding factor 3	894	2,86									17								
sp|Q6PJG2|EMSA1_HUMAN	ELM2 and SANT domain-containing protein 1	1045	3,31									17								
sp|Q96PK6|RBM14_HUMAN	RNA-binding protein 14	669	3,26				4,58				2,63	17				18				8
sp|Q9BRJ7|TIRR_HUMAN	Tudor-interacting repair regulator protein	211	2,91	6,02	6	6	4,39	10	4,72	4,03	8	16	35	32	27	20	63	25	21	48
sp|P31689|DNJA1_HUMAN	DnaJ homolog subfamily A member 1	397	2									16								
sp|P39023|RL3_HUMAN	60S ribosomal protein L3	403	3,18				4,48					16				24				
sp|Q02543|RL18A_HUMAN	60S ribosomal protein L18a	176	2,48				4					16				25				
sp|Q13310|PABP4_HUMAN	Polyadenylate-binding protein 4	644	2,52									16								
sp|P07437|TBB5_HUMAN	Tubulin beta chain	444	3,07			2	6	2				15			41	156	20			
sp|P29728|OAS2_HUMAN	2'-5'-oligoadenylate synthase 2	719	2,58									13								
sp|P62910|RL32_HUMAN	60S ribosomal protein L32	135	2									13								
sp|Q86V81|THOC4_HUMAN	THO complex subunit 4	257	2,04									13								
sp|Q9NX24|NHP2_HUMAN	H/ACA ribonucleoprotein complex subunit 2	153	2									13								
sp|Q9Y2W1|TR150_HUMAN	Thyroid hormone receptor-associated protein 3	955	1,41				5,42				2	13				37				8
sp|P37108|SRP14_HUMAN	Signal recognition particle 14 kDa protein	136	2									12								
sp|P62854|RS26_HUMAN	40S ribosomal protein S26	115	2,02									12								
sp|P22090|RS4Y1_HUMAN	40S ribosomal protein S4, Y isoform 1	263	2,14				4,62					11				24				
sp|P23246|SFPQ_HUMAN	Splicing factor, proline- and glutamine-rich	707	2,04									11								
sp|Q96KR1|ZFR_HUMAN	Zinc finger RNA-binding protein	1074	2									11								
sp|P15880|RS2_HUMAN	40S ribosomal protein S2	293	2									10								
sp|P18621|RL17_HUMAN	60S ribosomal protein L17	184	1,8									10								
sp|P47914|RL29_HUMAN	60S ribosomal protein L29	159	2,54				4,02					10				18				
sp|Q13151|ROA0_HUMAN	Heterogeneous nuclear ribonucleoprotein A0	305	2				4,1					10				22				
sp|Q15287|RNPS1_HUMAN	RNA-binding protein with serine-rich domain 1	305	2									10								
sp|Q92769|HDAC2_HUMAN	Histone deacetylase 2	488	2					2,01			2,03	10					10			7
sp|O95793|STAU1_HUMAN	Double-stranded RNA-binding protein Staufen homolog 1	577	2									9								
sp|P07910|HNRPC_HUMAN	Heterogeneous nuclear ribonucleoproteins C1/C2	306	1,77									9								
sp|P09651|ROA1_HUMAN	Heterogeneous nuclear ribonucleoprotein A1	372	2,07				9,48					9				48				

sp|P26368|U2AF2_HUMAN	Splicing factor U2AF 65 kDa subunit	475	1,84									9								
sp|P27635|RL10_HUMAN	60S ribosomal protein L10	214	2			1,47						9			7					
sp|P38919|IF4A3_HUMAN	Eukaryotic initiation factor 4A-III	411	1,42									9								
sp|P46013|KI67_HUMAN	Proliferation marker protein Ki-67	3256	1,4									9								
sp|P62899|RL31_HUMAN	60S ribosomal protein L31	125	1,85									9								
sp|P84098|RL19_HUMAN	60S ribosomal protein L19	196	1,49									9								
sp|Q5SSJ5|HP1B3_HUMAN	Heterochromatin protein 1-binding protein 3	553	1,72									9								
sp|P38646|GRP75_HUMAN	Stress-70 protein, mitochondrial	679	2,47	6,24	12		4,09	9,17	8,86		4	8	21	57		16	40	42		19
sp|P35637|FUS_HUMAN	RNA-binding protein FUS	526	1,38						4			8						18		
sp|Q13838|DX39B_HUMAN	Spliceosome RNA helicase DDX39B	428	2,61									8								
sp|Q1ED39|KNOP1_HUMAN	Lysine-rich nucleolar protein 1	458	1,43									8								
sp|Q96QC0|PP1RA_HUMAN	Serine/threonine-protein phosphatase 1 regulatory subun	940	1,57									8								
sp|Q9BY77|PDIP3_HUMAN	Polymerase delta-interacting protein 3	421	1,36									8								
sp|P14174|MIF_HUMAN	Macrophage migration inhibitory factor	115		4,01	6	4,04	4	2,04	4	3,39	2,03		75	61	60	19	42	67	17	37
sp|P62736|ACTA_HUMAN	Actin, aortic smooth muscle	377		1,7									45							
sp|Q6UWP8|SBSN_HUMAN	Suprabasin	590		6	6	4,6			4,58				30	32	44			32		
sp|P84103|SRSF3_HUMAN	Serine/arginine-rich splicing factor 3	164		3,33	2,03	2,08	4,38	2,78			2,41		30	8	8	35	14			11
sp|P62937|PPIA_HUMAN	Peptidyl-prolyl cis-trans isomerase A	165		4	4	2,24		4	6,92				24	28	10		20	33		
sp|P05109|S10A8_HUMAN	Protein S100-A8	93		4,59	8,13			2,01			8		19	51			8			35
sp|P21291|CSRP1_HUMAN	Cysteine and glycine-rich protein 1	193		2	2	2,1		2			2		18	15	15		12			10
sp|Q9HCC0|MCCB_HUMAN	Methylcrotonoyl-CoA carboxylase beta chain, mitochondr	563		4,53	12,39	4,97		3,07	10,99		4,93		16	66	20		14	62		18
sp|P68371|TBB4B_HUMAN	Tubulin beta-4B chain	445		3,83	2,09	8,3	19,85	4			5,39		14	12	54	171	19			37
sp|P06702|S10A9_HUMAN	Protein S100-A9	114		2	4			2			2		13	26			13			14
sp|P30048|PRDX3_HUMAN	Thioredoxin-dependent peroxide reductase, mitochondria	256		2,06	4			4			2		9	17			19			7
sp|P78406|RAE1L_HUMAN	mRNA export factor	368		2		1,39							9		9					
sp|Q96AB3|ISOC2_HUMAN	Isochorismatase domain-containing protein 2	205		2	2	2		2					8	11	7		7			
sp|P31025|LCN1_HUMAN	Lipocalin-1	176		2	2						2		8	8						9
sp|Q07666|KHDR1_HUMAN	KH domain-containing, RNA-binding, signal transduction-a	443		1,51				2,99			2,1		8				17			8
sp|Q9UKX2|MYH2_HUMAN	Myosin-2	1941		2,11									8							
sp|P62280|RS11_HUMAN	40S ribosomal protein S11	158		1,35	2								7	8						
sp|P26641|EF1G_HUMAN	Elongation factor 1-gamma	437		2	2,06								7	7						
sp|O43670|ZN207_HUMAN	BUB3-interacting and GLEBS motif-containing protein ZNF	478		1,6	2			2,01			2		6	9			9			9
sp|P04792|HSPB1_HUMAN	Heat shock protein beta-1	205		2	2,05	2							6	8	10					
sp|Q15717|ELAV1_HUMAN	ELAV-like protein 1	326		1,7							1,75		6							7
sp|Q99714|HCD2_HUMAN	3-hydroxyacyl-CoA dehydrogenase type-2	261			6					5				28					29	
sp|P04075|ALDOA_HUMAN	Fructose-bisphosphate aldolase A	364			6	2,99			5,44					27	17			26		
sp|P29508|SPB3_HUMAN	Serpin B3	390			4									16						
sp|P23528|COF1_HUMAN	Cofilin-1	166			2	4,16								14	17					
sp|P35080|PROF2_HUMAN	Profilin-2	140			2	2,11					2			14	17					9
sp|P47929|LEG7_HUMAN	Galectin-7	136			2									14						
sp|P18754|RCC1_HUMAN	Regulator of chromosome condensation	421			2						2			13						10
sp|Q5JST6|EFHC2_HUMAN	EF-hand domain-containing family member C2	749			2									8						
sp|Q86SX6|GLRX5_HUMAN	Glutaredoxin-related protein 5, mitochondrial	157			2									8						
sp|O95758|PTBP3_HUMAN	Polypyrimidine tract-binding protein 3	552			2									7						
sp|P15531|NDKA_HUMAN	Nucleoside diphosphate kinase A	152			2									7						
sp|P63173|RL38_HUMAN	60S ribosomal protein L38	70			2	2		2			2			7	8		11			6
sp|Q6P1X6|CH082_HUMAN	UPF0598 protein C8orf82	216			2	1,42								6	7					
cont|0031|GFP_AEQVI	Green fluorescent protein [Aequorea victoria (contaminar	238				13,87	11,15	14,8	14,79	14,05	14,89				120	77	145	137	162	156

sp|P37275|ZEB1_HUMAN	Zinc finger E-box-binding homeobox 1	1124	12,75	10,71	12,15	10,59	12,3	13,26	111	132	203	157	216	224
sp|P78385|KRT83_HUMAN	Keratin, type II cuticular Hb3	493	2,8						45					
sp|P0DPA2|VSIG8_HUMAN	V-set and immunoglobulin domain-containing protein 8	414	5,93						33					
sp|P0CG48|UBC_HUMAN	Polyubiquitin-C	685	5,39	5,04	2,47			4	27	22	11			15
sp|P01040|CYTA_HUMAN	Cystatin-A	98	4,13						24					
sp|P10599|THIO_HUMAN	Thioredoxin	105	4						20					
sp|P0DMV8|HS71A_HUMAN	Heat shock 70 kDa protein 1A	641	3,1						15					
sp|P31151|S10A7_HUMAN	Protein S100-A7	101	2,09						12					
sp|P04080|CYTB_HUMAN	Cystatin-B	98	2						10					
sp|P07237|PDIA1_HUMAN	Protein disulfide-isomerase	508	2						10					
sp|P09874|PARP1_HUMAN	Poly [ADP-ribose] polymerase 1	1014	2						10					
sp|P07355|ANXA2_HUMAN	Annexin A2	339	2,09		1,13			5,54	9		7			23
sp|P17931|LEG3_HUMAN	Galectin-3	250	2						8					
sp|Q13523|PRP4B_HUMAN	Serine/threonine-protein kinase PRP4 homolog	1007	1,8						8					
sp|P29372|3MG_HUMAN	DNA-3-methyladenine glycosylase	298	2		2,01			2	7		10			7
sp|Q9H910|JUPI2_HUMAN	Jupiter microtubule associated homolog 2	190	1,48						6					
sp|Q07955|SRSF1_HUMAN	Serine/arginine-rich splicing factor 1	248		5,59						41				
sp|O43390|HNRPR_HUMAN	Heterogeneous nuclear ribonucleoprotein R	633		8,96						34				
sp|P62829|RL23_HUMAN	60S ribosomal protein L23	140		4,2						30				
sp|P38159|RBMX_HUMAN	RNA-binding motif protein, X chromosome	391		6						29				
sp|Q92804|RBP56_HUMAN	TATA-binding protein-associated factor 2N	592		4						25				
sp|P56545|CTBP2_HUMAN	C-terminal-binding protein 2	445		4,56	2,14		7,42	4,04		22	10		52	18
sp|P46776|RL27A_HUMAN	60S ribosomal protein L27a	148		4						21				
sp|P52597|HNRPF_HUMAN	Heterogeneous nuclear ribonucleoprotein F	415			2			2,34			41			44
sp|Q15366|PCBP2_HUMAN	Poly(rC)-binding protein 2	365			2,7						13			
sp|Q9ULA0|DNPEP_HUMAN	Aspartyl aminopeptidase	475			1,72		12,76				9		69	
sp|O75912|DGKI_HUMAN	Diacylglycerol kinase iota	1065			1,19						8			
sp|Q9UQ35|SRRM2_HUMAN	Serine/arginine repetitive matrix protein 2	2752			1,8						8			
sp|P54886|P5CS_HUMAN	Delta-1-pyrroline-5-carboxylate synthase	795			1,42						7			
sp|P55317|FOXA1_HUMAN	Hepatocyte nuclear factor 3-alpha	472			2			2			7			10
sp|Q15084|PDIA6_HUMAN	Protein disulfide-isomerase A6	440					15,79						112	
sp|P69905|HBA_HUMAN	Hemoglobin subunit alpha	142						2						12
sp|P15924|DESP_HUMAN	Desmoplakin	2871						2						9
sp|P01111|RASN_HUMAN	GTPase NRas	189						2						8
sp|P68871|HBB_HUMAN	Hemoglobin subunit beta	147						2						8
